# Laparoscopic sleeve gastrectomy in severely obese adolescents: effects on metabolic profile

**DOI:** 10.1590/2359-3997000000310

**Published:** 2017-12-01

**Authors:** Ruth Rocha Franco, Marina Ybarra, Louise Cominato, Larissa Mattar, Leandra Steinmetz, Durval Damiani, Manoel Carlos Prieto Velhote

**Affiliations:** 1 Universidade de São Paulo Faculdade de Medicina Departamento de Endocrinologia Pediátrica São Paulo SP Brasil Departamento de Endocrinologia Pediátrica do Instituto da Criança do Hospital das Clínicas da Faculdade de Medicina da Universidade de São Paulo (ICr-HCFMUSP), São Paulo, SP Brasil; 2 Universidade de São Paulo Faculdade de Medicina Departamento de Nutrição Pediátrica São Paulo SP Brasil Departamento de Nutrição Pediátrica do Instituto da Criança do Hospital das Clínicas da Faculdade de Medicina da Universidade de São Paulo (ICr-HCFMUSP), São Paulo, SP, Brasil; 3 Universidade de São Paulo Faculdade de Medicina Departamento de Cirurgia Pediátrica São Paulo SP Brasil Departamento de Cirurgia Pediátrica do Instituto da Criança do Hospital das Clínicas da Faculdade de Medicina da Universidade de São Paulo (ICr-HCFMUSP), São Paulo, SP, Brasil

**Keywords:** Bariatric surgery, sleeve gastrectomy, obesity, adolescent

## Abstract

**Objective:**

The objective was to conduct clinical and metabolic evaluations of obese adolescents before and after laparoscopic sleeve gastrectomy (LSG) (up to 24 months).

**Subjects and methods:**

This was designed as a retrospective, descriptive series of cases study, conducted in Instituto da Criança, São Paulo, Brazil. Analysis of clinical and laboratory data from 22 obese adolescents between 14 and 19 years old submitted to LSG between 2007 and 2014. Patients had BMI > 40 kg/m^2^ or BMI > 35 kg/m^2^ with comorbidities. Anthropometric, clinical and laboratory assessments were performed: before surgery, 6, 12, 18, and 24 months after surgery. We assessed weight loss and metabolic changes up to 24 months after LSG.

**Results:**

The mean preoperative weight and BMI were 128.5 kg (SD = 23.1) and 46.5 kg/m^2^ (SD = 74), respectively. There was an average weight loss of 34.5 kg in the first 12 months’ post LSG, corresponding to a 60% excess weight loss (EWL), as well as an average reduction in BMI of 12.3 kg/m^2^. However, after 24 months, the average EWL was 45%, corresponding to an average weight regain (WR) of 13.3 kg (15%) within two years. LSG improved dyslipidemia in 67.8% of patients, a significant remission of hepatic steatosis 47% and 37.7% systemic arterial hypertension; type 2 diabetes remission was complete.

**Conclusions:**

LSG proved to be a safe and effective procedure and seems to be the new hope for the obesity epidemic.

## INTRODUCTION

As a chronic and progressive disease, obesity is currently considered a global epidemic that causes 2.8 million deaths per year. The prevalence of overweight and obese children have increased worldwide, with an estimate of 60 million obese children in 2020 (1).

The risk of becoming an obese adult is 77% for obese children and 7% for non-obese children, and it has been shown that 7.3% of boys and 5.5% of girls in the United States are extremely obese (BMI ≥ 35 kg/m^2^ ≥ or BMI ≥ 1.2 above the 95^th^ percentile) (2,3). In Brazil, data from the Family Expenditure Survey 2008-2009 conducted by the Brazilian Institute for Geography and Statistics showed a significant increase of overweight children, mainly in the age group between 5 and 9 years old. The number of obese children within the same age range increased by over 300%, jumping from 4.1% in 1989 to 16.6% in 2009. The number of overweight boys more than doubled between 1989 and 2009, rising from 15% to 34.8%. Among girls, this variation was even greater: 2.4 in 1989 to 11.8 in 2009 (4).

The metabolic risks faced by obese children and adolescents, as well as the related comorbidities, are widely known (5). In obese adolescents with diabetes, weight loss can improve glycemic control, prevent the development of type 2 diabetes mellitus (type 2 DM) in pre-diabetic teens, reduce cardiovascular risk and improve quality of life (6).

However, clinical, pharmacological and behavioral treatments have had disappointing outcomes in severely obese adults, adolescents and children. The consensus is that in adults, advanced stages of obesity only respond satisfactorily to surgical treatments, and surgical treatment for obesity in adolescents has recently been gaining acceptance (7). The current guidelines of the International Pediatric Endosurgery Group (IPEG) recommend that surgical intervention should be considered only for extremely obese adolescents. Complete remission of type 2 DM in adolescents submitted to bariatric surgery has also been reported (8).

A meta-analysis done in 2008 included 19 studies on bariatric surgery in obese adolescents with a mean age of 16.8 years and mean body mass index (BMI) of 48.8 kg/m^2^ showed a significant reduction in BMI when patients underwent a bypass or gastric banding (7). Lately, less invasive techniques have been proposed for the pediatric age group with published efficacy and safety (9,10). Since 2007 laparoscopic sleeve gastrectomy (LSG) is one of these procedures. Performing LSG in this age group allows for intervention before the comorbidities become more severe.

LSG was initially used as part of the biliopancreatic bypass with duodenal switch (BPD-DS) (11). Later, in difficult cases, the procedure was performed in two stages and surprisingly good results were observed with the LSG alone, in spite of minimal restriction and malabsorption (12). Currently, it is an isolated procedure among the arsenal of surgical procedures. LSG is a relatively simple procedure, with low morbidity and mortality, and the ample literature on it shows that it can lead to loss of excess weight in a range of 5461% without device implantation or dissociation of the gastrointestinal tract (13).

There is still a lack of consensus regarding the inclusion criteria for obese adolescents in surgical obesity treatment programs, what type of surgery would be most appropriate for this population, and how postoperative follow-up should be conducted. One of the most serious medium and long-term problems is weight regain, which varies across bariatric surgery patients (14,15). Little data exist on the metabolic changes after bariatric surgery in adolescents.

The goal of this study was to conduct clinical and metabolic evaluations of obese adolescents before and after LSG during a period of 24 months in order to obtain a clearer picture of postsurgical outcomes and better understand the subgroup of patients who might benefit from this procedure.

## SUBJECTS AND METHODS

This is a retrospective, descriptive series of cases study. Inclusion criteria was defined as patients with age from 14 to 19 years old, had BMI ≥ 40 kg/m^2^ or BMI ≥ 35 kg/m^2^ with comorbidities and were submitted to LSG between 2007 and 2014 at *Instituto da Criança da Universidade de São Paulo* (Children's Institute of the University of São Paulo) Exclusion criteria were patients with no follow-up data available.

We conducted the analysis of a clinical and laboratory data. All patients attended the child obesity outpatient clinic of the Pediatric Endocrinology Department. The follow-up consists of clinical and pharmacological treatment. It includes a clinical appointment once a month for at least 6 months before surgery, followed by a multidisciplinary team including a pediatric endocrinologist, a nutritionist, a psychologist and a physical educator. Clinical treatment was composed of guidance on the lifestyle, diet and physical activity. Pharmacological treatment included the use when indicated of metformine, sibutramine and anti-depressive drugs as fluoxetine and sertraline. LSG was recommended to patients who failed to achieve significant weight loss (10% of initial weight at 6 months) through clinical treatment. Both the patients and their guardians were informed about the risks and benefits of surgery and provided informed consent.

Anthropometric data such as weight (kg), height (m) and BMI (kg/m^2^) as well as abdominal circumference (AC) were retrieved from medical records. Weight loss and reductions in BMI were reported in absolute values and as a percentage of the initial values. Excess Weight Loss (EWL) was measured using BMI values above 25 kg/m^2^.

Clinical and laboratory assessments were performed during the following times: before surgery, and 6, 12, 18, and 24 months after surgery. We evaluated: total cholesterol (TC), fractions [Low-density lipoprotein (LDL-C), high-density lipoprotein (HDL-C) and triglycerides (TG) (colorimetric enzyme, mg/dL)], oral glucose tolerance test (OGTT) with oral administration of 75g of glucose, fasting glycaemia (FG) (enzymatic colorimetric, mg/dL) and fasting insulinemia (electrochemiluminescence immunoassay, μU/mL), glycated hemoglobin (Hb) (ion exchange high performance liquid chromatography HPLC - Variant II Turbo - method certified by NGSP), transaminases (kinetic UV - IFCC, U/L), uric acid (enzymatic colorimetric assay, mg/dL), abdominal ultrasonography for evaluation of hepatic steatosis and echocardiogram to evaluate concentric hypertrophy of the left ventricle.

We evaluated the following variables: insulin resistance (IR; using the homeostatic model assessment of insulin resistance, HOMA-IR ≥ 2.5), pre-diabetes (FG ≥ 100 mg/dL and < 126 mg/dL or OGTT ≥ 140 and < 200 mg/dL), type 2 DM (FG ≥ 126 mg/dL or OGTT ≥ 200 mg/dL or glycated Hb ≥ 6.5%), dyslipidemia (TC > 200 or LDL-C > 130 or HDL-C < 40 for boys and HDL-C < 45 for girls or TG > 130 mg/dL), and systemic arterial hypertension (SAH) [systolic blood pressure > 130 mmHg or diastolic blood pressure > 80 mmHg]. The resolution of comorbidities was evaluated throughout follow-up. Metabolic Syndrome was considered when any 3 of 5 were present: elevated waist circumference (> 102 cm in men; > 88 cm in women), elevated triglycerides (≥ 150 mg/dL), reduced HDL-C (< 40 mg/dL in men; < 50 mg/dL in women), elevated blood pressure (≥ 130 mmHg systolic blood pressure or ≥ 85 mmHg diastolic blood pressure), elevated fasting glucose >100 mg/dL (16).

Surgical descriptions, including complications, were retrieved from the medical records. After surgery, patients were followed by the same multidisciplinary team including a pediatric endocrinologist, a nutritionist, a psychologist and a physical educator. Physical activity was encouraged and all followed a one year diet with a nutritionist who introduced gradually each type of food according to a LSG guideline (17,18) and routinely received multivitamin, vitamin B1 and vitamin D3 supplements. The LSG technique has been well standardized (14).

Means were calculated for every period studied. We used a repeated measures model using generalized estimating equations (GEE) to calculate the effect over time considering a normal distribution of the response variables and the autoregressive working correlation matrix. We present the p-values for the comparisons in pairs, comparing each time to the baseline time and then to the time immediately before it. Significance was set at 5%. All analyses were conducted using the *ggplot2* and *geeglm* packages of the R 3.1.1 software (R Core Team).

## RESULTS

We assessed 22 obese adolescents (16 females) with a mean age of 16.89 years). The mean preoperative weight and BMI were 128.5 kg (SD 23.1) and 46.5 kg/m^2^ (SD 7.4), respectively. The average operation (anesthesia and surgery) time was 256 minutes. There were no open conversions or postoperative complications. One patient had a spleen injury and another had intraoperative port site bleeding. The mean hospital stay was four days (considering that patients were admitted one day before the surgery), without any readmissions or deaths. The average number of postoperative appointments was 8.9 (SD 4.0) during an average of 27.6 months of follow-up.

**Figure 1:**
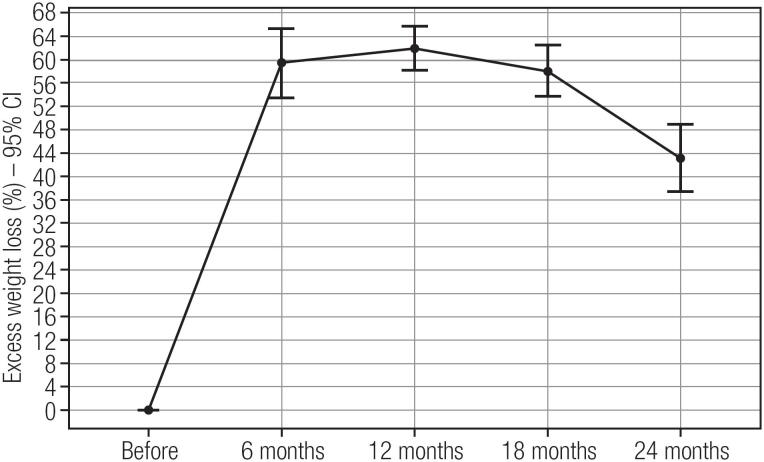
Percent of excess weight loss (EWL) after laparoscopic sleeve gastrectomy in severely obese adolescents.

**Figure 2:**
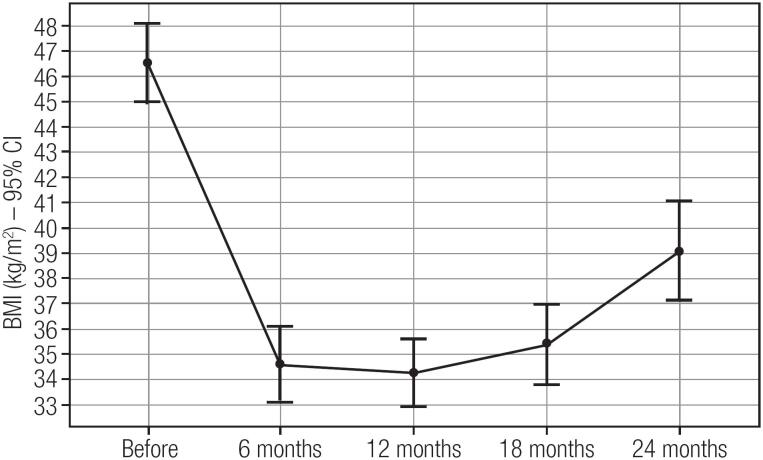
Percent of reduction in body mass index (BMI) after laparoscopic sleeve gastrectomy in severely obese adolescents.

There was an average weight loss of 34.5 kg in the first 12 months’ post LSG, corresponding to a 60% EWL, as well as an average reduction in BMI of 12.3 kg/m^2^. However, after 24 months, the average EWL was 45%, corresponding to an average weight regain (WR) of 13.3 kg (Figures 1 and 2).

**Figure 3:**
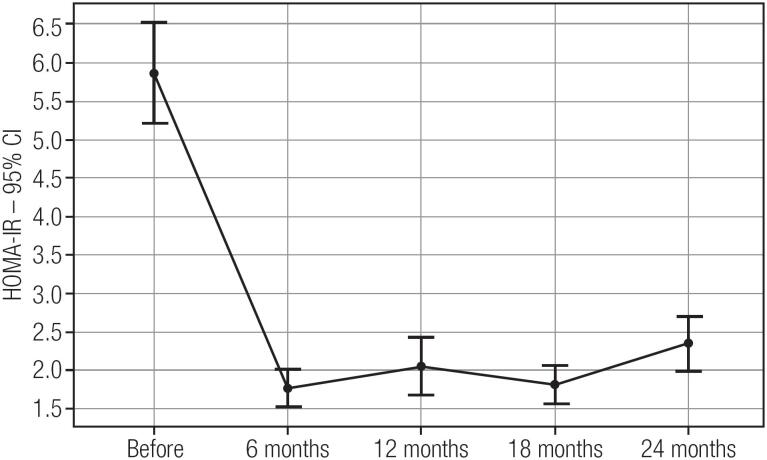
Mean HOMA-IR (homeostatic model assessment of insulin resistance) post laparoscopic sleeve gastrectomy in severely obese adolescents.

Before surgery, more than half of the patients were hypertensive and had hepatic steatosis, and two had concentric hypertrophy of the left ventricle. The baseline data and the following months data are shown in Table 1. There were also high rates of dyslipidemia and IR (Figure 3). Note that after LSG all the comorbidities remitted or improved (Table 2). Twelve months after LSG, all metabolic markers remained stable and there was no weight regain. The course of metabolic syndrome before and after surgery is also shown in Table 2.

**Table 1:** Comparison of the baseline characteristics and the evolution after 6, 12, 18 and 24 months

Variables	Baseline		6 months		12 months		18 months		24 months	
	N	Mean (SD)	N	Mean (SD)	N	Mean (SD)	N	Mean (SD)	N	Mean (SD)
BMI (kg/m^2^)	23	46.3 (7.4)	21	34.4 (6.6)* †	15	34.5 (6.4)*	12	37.1 (6.2)*	14	38.4 (6.7)
WC (cm)	19	133.6 (13.7)	19	102.7 (27)* †	14	108.4 (13.8)*	10	114.4 (15.2)	13	116.6 (13.8)
SBP (mmHg)	23	127.8 (12.5)	19	113.7 (10.4)* †	14	119.7 (15.4)	8	113.9 (8.4)	14	120.1 (7.8)
DBP (mmHg)	23	80.2 (9.9)	19	69.8 (6.4)	14	72.2 (9.2)	8	70 (3.1)	14	75.1 (8.7)†
TC (mg/dL)	21	163.3 (25)	13	149.7 (15.3)	9	153.7 (23.7)	7	135.2 (10.6)	8	155.8 (25.6)
LDL-C (mg/dL)	20	106 (21.8)	13	94.1 (18)	9	91.4 (24.6)	7	79.9 (12.3)*	8	96.2 (23.4)
HDL-C (mg/dL)	20	36.6 (10.9)	13	42.3 (8)* †	9	51.4 (8.4)* †	7	48.4 (12.8)*	8	50.6 (14.1)*
TG (mg/dL)	21	115.6 (52.4)	13	91.8 (38.8)	9	68.7 (18.8)*	7	84.6 (31.1)*	8	90.5 (43.3)
Glycaemia (mg/dL)	19	59.5 (44.3)	13	66.6 (30.6)*	9	49 (38.7)	5	60.9 (37.1)	9	80.3 (6.8)*
Insulinemia (μU/mL)	11	38.6 (29.8)	10	12.1 (9.4)	6	8.4 (3.1)*	5	11 (8.9)	8	12.1 (8.2)

BMI: body mass index; WC: waist circumference; SBP: systolic blood pressure; DBP: diastolic blood pressure; TC: total cholesterol; LDL-C: low-density lipoprotein; HDL-C: high-density lipoprotein; TG: triglycerides; SD: standard deviation; N: number

* statistic significant when compared to 6-month mean

† statistic significant when compared to the mean immediately before.

**Table 2:** Prevalence of comorbidities at baseline and after 12 and 24 months of laparoscopic sleeve gastrectomy (LSG) in severely obese adolescents

Measure	Baseline	12 months	24 months	p value
SAH	13/22 (59.1%)	3/17 (17.6%)	3/14 (21.4%)	0.023
type 2 DM	1/22 (4.5%)	0/12 (0%)	0/13 (0%)	0.999
OGI	2/22 (9.1%)	0/12 (0%)	0/13 (0%)	0.999
LV hypertrophy	3/22 (13,6%)	0/12 (0%)	0/12 (0%)	0,999
HOMA-IR > 2.5	21/22 (95.5%)	4/12 (33.3%)	6/13 (46.2%)	0.046
Hepatic steatosis	12/22 (54.5%)	2/12 (16.7%)	1/14 (7.1%)	0.027
Dyslipidemia	21/22 (95.5%)	2/12 (16.7%)	3/13 (23.1%)	0.004
Metabolic syndrome	4/22 (18.2%)	4/22 (18.2%)	1/14 (7.1%)	0.479

SAH: systemic arterial hypertension; type 2 DM: type 2 diabetes; OGI: oral glucose intolerance; LV hypertrophy: left ventricular hypertrophy; HOMA-IR: homeostatic model assessment of insulin resistance.

Throughout the postoperative follow-up, there were no deficiencies in vitamin D, albumin, vitamin B12, folic acid, calcium, magnesium, phosphorus or zinc dosages (data not shown). One female patient presented iron deficiency anemia with low concentrations of iron and ferritin. No patients developed a compulsive behavior. Twelve patients underwent bone densitometry (BD) 15 months after LGV, on average. Everyone showed normal BD for age and sex.

## DISCUSSION

Considering the low response to clinical treatments and the lowered life expectancy of severely obese adolescents, bariatric surgery seems to be the new hope for the obesity epidemic. However, the long-term implications of surgery, such as psychological effects, metabolic interference and the impact on growth are not yet fully understood.

In this study, we conducted clinical and metabolic evaluations of obese adolescents before and after LSG during a period of at least 24 months. In our group of 22 adolescents, we observed a mean weight loss of 34.5 kg (average excess weight loss of 60% - EWL) at 12 months. After 24 months, the average weight loss was 25.8 kg (45% EWL), which means 15% of the weight regain within two years. This is similar to that reported by Nadler and cols., who evaluated 33 patients with an average EWL of 40 ± 19% after 12 months (19). On the other hand, our values were lower than those reported by Alqahtani and cols., who studied a group of 108 patients with a mean EWL of 64% at 24 months (9) and Boza and cols., who studied 54 patients and recorded a mean EWL of 96.2% after 12 months (20). From our experience this weight regain is due to a lack of compromise with diet and exercise prescription after the first year of follow-up, although we have no precise evaluation concerning this topic in this study.

While we found a 25.6% weight loss at 12 months, Sachdev and cols. similarly reported a loss of 27% (21). Our data were also like those of Lennerz and cols., who observed a loss of 13.1 ± 8.2 BMI points at 18 months’ post-surgery, while in our study this figure was 11.1 (22).

While WR was 15% in our study, Boza and cols. reported a WR of only 4% in 2 years and Alqahtani and cols. did not report any (9,20). The lowest weight point in our study was 12 months, in line with studies reporting 12-16 months (23). WR is a common risk, and approximately 20-30% of patients do not reach their ideal weight loss (24,25).

Several factors can influence weight goals and WR after bariatric surgery. Responses vary between individuals according to type of surgery, follow-up, demographics, psychosocial factors, biological factors and factors that regulate energy intake, stock and expenditure (25).

Our data showed a rapid decrease in blood glucose and a significant improvement in insulin sensitivity already at 6 months postoperatively, when there was still a mean BMI of 40.1 kg/m^2^ (SD 5.8). Previous studies related the different types of bariatric surgery with improved glucose homeostasis together with incretins and intestinal hormones, independently of weight loss (6,26-29). This was observed when they compared weight loss by bariatric surgery to weight loss achieved with clinical treatments or purely restrictive treatments such as gastric banding, which have little or no effect on the post-prandial hormonal profile (30-32).

Bariatric surgery not only reduces body fat; it also improves dyslipidemia. A study that evaluated a cohort of patients who had bariatric surgery showed improved serum lipid profiles in 70% of patients (33). In our study, dyslipidemia improved in 67.8% of patients.

Still regarding comorbidities, our study revealed a significant resolution rate in two years, with approximately 47% remission of hepatic steatosis and 37.7% of SAH. Type 2 DM remission was complete. Of the 95.5% of patients with IR preoperatively, 66.6% normalized their insulin profile and kept it despite the weight regained in two years. The significant improvement in dyslipidemia also remained despite the weight regained. This shows that the metabolic condition achieved by LSG goes beyond simple weight loss, which makes the surgery more metabolic in nature and not purely restrictive, as formerly thought. Indeed, the International Hepatology Committee considers bariatric surgery a valid treatment option for non-alcoholic steatohepatitis in adolescents with morbid obesity (34).

Limitations of our study are that it was a retrospective series of cases study with a small number of patients, no control group and non-standard follow-up. The same surgeon with the same surgical technique operated all patients and all exams were performed in the same laboratory.

The increase in the number of patients undergoing bariatric surgery has allowed for a better understanding of the mechanisms that induce weight loss. However, it is not yet clear which physiological mechanisms are responsible for continued weight loss and metabolic improvements (35). This work shows that even with weight regain, patients show great metabolic improvement, which is maintained for up to two years after surgery. Prospective studies should be conducted comparing different surgical treatments using standardized long-term follow-ups to address these issues.
